# CT-guidance interstitial ^125^Iodine seed brachytherapy as a salvage therapy for recurrent spinal primary tumors

**DOI:** 10.1186/s13014-014-0301-8

**Published:** 2014-12-23

**Authors:** Qianqian Cao, Hao Wang, Na Meng, Yuliang Jiang, Ping Jiang, Yang Gao, Suqing Tian, Chen Liu, Ruijie Yang, Junjie Wang, Kaixian Zhang

**Affiliations:** Department of Radiation Oncology, Cancer center, Peking University Third Hospital, No. 49 North Garden road, Haidian district Beijing, 100191 China; Department of Radiology, Peking University Third Hospital, No. 49 North Garden road, Haidian district Beijing, 100191 China; Cancer Center, Tengzhou Central People’s Hospital, Tengzhou city, Shangdong Province 277500 China

**Keywords:** Brachytherapy, ^125^I seed implantation, Primary spine tumors, CT-guided, Outcome

## Abstract

**Background:**

Management of spinal neoplasms has relied on open surgery and external beam radiotherapy (EBRT). Although primary spinal tumors are rare, their treatment remains a pervasive problem. This analysis sought to evaluate the safety and efficacy of CT-guided ^125^I seed brachytherapy for recurrent paraspinous and vertebral primary tumors.

**Methods:**

From November 2002 to June 2014, 17 patients who met the inclusion criteria were retrospectively reviewed. 14 (82.4%) had previously undergone surgery, 15 (88.2%) had received conventional EBRT and 3 (17.6%) had chosen chemotherapy. The number of ^125^I seeds implanted ranged from 7 to 122 (median 79) with specific activity of 0.5-0.8 mCi (median 0.7 mCi). The post-plan showed that the actuarial D_90_ of ^125^I seeds were 90–183 Gy (median 137 Gy). The follow-up period ranged from 2 to 69 months (median 19 months). The local control rate was calculated by the Kaplan-Meier method.

**Results:**

For 5 Chondrosarcomas, the 1-, 2-, 3-year local control rates were 75%, 37.5%, and 37.5%, respectively, with a median of 34 months (range, 4–39 months). For 4 chordomas, the local control rate was 50% with a median follow-up of 13 months (range, 3–17 months). For 3 fibromatosis, all of them were survival without local recurrence at the end of follow-up. During the follow-up period, 35.3% (6/17) died from metastases, 17.6% (3/17) developed local recurrence by 8, 14 and 34 months while 64.7% (11/17) remained alive. 100% experienced pain relief and normal or improved ambulation, without more than Frankel grade 3 radiation myelopathy.

**Conclusions:**

Percutaneous ^125^I seed implantation can be an alternative or retreatment for recurrent spinal primary tumors.

## Background

Primary spinal tumors which can be benign or malignant are relatively uncommon compared with metastases to the spine [1]. Common benign tumors in the spine include osteoid osteoma, osteoblastoma, aneurysmal bone cysts, osteochondroma and giant cell tumor. Malignant processes that can develop in the spine include multiple myeloma, osteosarcoma, chondrosarcoma, plasmacytoma and Ewing’s sarcoma. These various types of tumor result in a broad spectrum of symptoms ranging from backache to paraplegia. Initial presenting symptoms may include neural dysfunction, local pain or spinal deformation. Given the complex structure of the spine, the biological characteristics vary among different malignancies, leading to differing sensitivities to chemotherapy and EBRT [2]. To deliver appropriate treatment, it is important that radiation therapists understand each type of neoplasm.

Primary paraspinous and vertebral column tumors are relatively uncommon and their treatment is a challenging problem due to the anatomic location and organs at risk [2]. Traditionally, the gold standard treatment for primary spinal tumors has been open surgical excision, though corporectomy and spondylectomy are not wide anatomical resection indeed [3,4]. EBRT also plays a prominent role because of the significant side effects and disadvantages of surgery [5,6]. However, due to coexisting medical problems or unacceptable surgical complication risks, many patients are not candidates for surgery or EBRT [7]. A treatment modality with improved outcomes, good tolerability and a short hospital stay is therefore needed.

CT-guided ^125^I seed implantation therapy has been the focus of recent studies that have demonstrated excellent clinical outcomes [8], but has rarely been reported in the treatment of recurrent spinal primary tumors [9]. Here we report our initial results with ^125^I seed brachytherapy for recurrent paraspinous and vertebral column tumors and discuss the possible advantages of this technology.

## Materials and methods

### Characteristics of patients

In this study, we retrospectively reviewed 17 patients who were treated at our department between November 2002 and June 2014. There were 13 men and 4 women with a median age of 52 years (range 20–71 years). 5 had chondrosarcoma, 4 were chordoma, 3 fibromatosis and the last 5 were paraganglioma, lymphoma, osteosarcoma, malignant giant cell tumor and vertebral hemangioma, respectively (Table 1). The tumors involved the arch as well as the vertebral body in 35.3% patients, while 64.7% involved the vertebral body alone. Before undergoing interstitial ^125^I brachytherapy, all 17 patients had previously undergone treatment to the spine, 14 had undergone corporectomy or spondylectomy and tumor resection, 15 had received EBRT (the total cumulative doses of 39.6-78 Gy, with a median of 54 Gy) and 3 had received chemotherapy (Table 2). All patients were normal for heart, liver, kidney and blood functional markers and showed no signs of infection. Life expectancy was more than 6 months.

**Table 1:** **Patients characteristics (n = 17)**

	**No. of patients**	**Percentage (%)**
Median age	52 (20-71)	
Gender		
Male	13	76.5
Female	4	23.5
KPS		
60	5	29.4
70	5	29.4
80	2	11.8
90	4	23.5
100	1	5.9
Tumor pathology		
Chondrosarcoma	5	29.4
Chordoma	4	23.5
Fibromatosis	3	17.6
Osteosarcoma	1	5.9
Lymphoma	1	5.9
Paraganglioma	1	5.9
Malignant giant cell tumor	1	5.9
Malignant vertebral hemangioma	1	5.9
Previous surgery	14	82.4
Previous EBRT	15	88.2
Previous chemotherapy	3	17.6
Follow-up (months)		
Median (months) (range)	19 (2-69)

**Table 2:** **General information of patients before** ^**125**^ **I seed implantation (n = 17)**

**No.**	**Gender**	**Age**	**Pathological diagnosis**	**Location**	**Pre-seed implant therapy**	**PS:NRS**	**ASIA**	**Ambulatory function score**
1	Male	59	Chordoma	S3	S+RT60Gy	5-6	E	
2	Male	69	Chondrosarcoma	T9-11	S	4	B	
3	Male	50	Chondrosarcoma	L2-S1	S+RT60Gy	8-9	C	
4	Male	50	Parganglioma	L1-L2	S+RT46Gy	4-6	C	
5	Male	71	Chondrosarcoma	C2-5	S	3	D	
6	Male	45	Chondrosarcoma	L3,4	S+RT64Gy+CTx	4-5	D	
7	Female	20	Lymphoma	S1-5	CTx+RT54Gy	6	C	
8	Male	46	Osteosarcoma	S1-5	CTx+RT54Gy	4	E	
9	Female	57	Fibromatosis	C3-T2	RT60Gy	3	E	
10	Male	47	Chondrosarcoma	C4-T3	S+RT64Gy	3-4	D	
11	Male	69	Chordoma	S3	S+RT50Gy	4	E	
12	Male	52	Chordoma	C2-4	S+RT44Gy	1	D	
13	Female	57	Chordoma	C2	S+RT40Gy	2	D	
14	Female	46	Fibromatosis	T1-3	S+RT78Gy	2	D	
15	Female	68	Malignant giant cell tumor	T1	S+RT39.6Gy	6	C	
16	Male	56	Fibromatosis	C5	S+RT50Gy	4	C	
17	Male	52	Malignant vertebral hemangioma	C2	S+RT40Gy+CTx	5-7	C	

Before administration of the radioisotope, informed signed consent was obtained in accordance with our quality assurance program. The study followed the guidelines for experimental investigation with human subjects required by our institution. This retrospective research has been approved by the ethics committee in Peking University 3rd Hospital and carried out in accordance with the Helsinki Declaration [10]. The inclusion criteria for the study were: Karnofsky performance status (KPS) >60; definite pathological diagnosis; isolated or limited number of malignant tumors;recurrent or residual spinal tumors after irradiation and/or chemotherapy; residual tumors after surgery resection; for both malignant and benign paraspinal lesions, patients underwent seed implantation because they refused or their status did not allow surgery or chemoradiotherapy; tumor identified on CT before implantation; and follow-up until death. Exclusion criteria were as follows: poor general health or serious medical condition present that meant the patient could not tolerate implantation; poor coagulation function; radiation resistance; and no proper path for the needles.

### Preparation of seed implants

Many specialized devices have been developed to increase the precision and accuracy of dose delivery. To determine accurately the seeds’ position, CT guidance was used (64 slice spiral; Lightspeed VCT; GE Healthcare, Piscataway, NJ). Radioactive ^125^I seeds were provided by the China Institute of Atomic Energy (model 6711; Beijing Atom and High Technique Industries Inc., Beijing, China), of size 0.8 mm × 4.5 mm and activity 0.50-0.80 mCi. For implantation, 18G needles and a Mick applicator (Mick Radio-Nuclear Instruments Inc., Mount Vernon, NY) were used. The radiation oncologist contoured the gross tumor volume (GTV) on each transverse image, the planning target volume (PTV) included 0.5-1.0 cm of GTV peripheral tissue. A three-dimensional (3D) EBRT planning system (Beijing Fei Tian Industries Inc., Beijing, China) was used to reconstruct 3D images of the tumor and calculate the number and dose rate distribution of ^125^I seeds. The D_90_ (the doses delivered to 90% of the target volume defined by CT using dose-volume histogram) was 90–183 Gy (median 137 Gy, over the period of total decay) and the number of seeds per patient ranged from 7–122 (median 79). The treatment efficacy, with regard to dose validation, was evaluated from a dose-volume histogram after the procedure.

### Treatment protocols

During the procedure, patients remained in a prone position to facilitate CT guidance. We measured the pedicle angle and the distance of the puncture point from the spinous process, which was maintained at 1.0 cm. The depth of the puncture point to the pedicle was also borne in mind. After administration of local anesthesia, for cervical, thoracic and lumbar neoplasms, needles were inserted through the anterior portion of the lateral images, in the interval between the pedicle and the vertebral body, or with a pedicle or paraspinal approach, respectively. Radioactive seeds were implanted in accordance with the principles of Paris [11], in a linear arrangement, parallel and 0.5-1.0 cm apart. Seeds were implanted 1.0 cm from the spinal cord, to minimize radiation damage. Immediately after the procedure, a CT scan was obtained to confirm the location of the seeds in case of any need for supplementary implantation. Patients remained in bed for 6 h with routine hemostatic treatment and antibiotic prophylaxis, and their vital signs were observed.

### Follow-up and statistical analyses

Tumor response was initially evaluated clinically and radiologically at 4 weeks and thereafter at intervals of 2 months for the first year and every 6 months thereafter. Local control time was measured from the beginning of therapy. We used a numeric rating scale (NRS) to measure pain scores as follows: 0, no pain; 1–3, mild pain; 4–6, moderate pain causing insomnia; 7–10, severe pain. Frankel was used for measuring radiation myelopathy. American Spinal Injury Association (ASIA) grade was applied for neurological assessment. The Radiation Therapy Oncology Group/European Organisation for Research and Treatment of Cancer radiation injury grading was used to evaluate side effects after implantation. Local control rates were generated by the Kaplan-Meier method and SPSS 18.0 (IBM, Armonk, NY) was used for statistical analysis.

## Results

### Treatment efficacy

17 patients (100%) were followed-up, with a median follow-up time of 19 months (range 2–69 months). Symptom and local control were achieved in all 17 patients (100%). None was lost to follow-up. All cases were assessed clinically and with imaging until the follow-up time or death.

**Figure 1:**
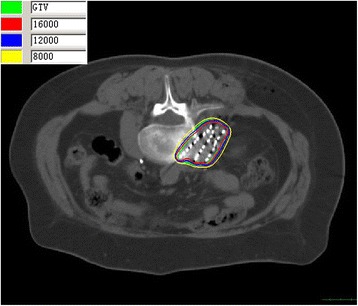
**The isodose curve distribution of tumor after seed implantation from CT scan.** The inner green curve represents GTV. The ellipses are isodose lines of 160, 120, 80 Gy from inside, respectively.

**Figure 2:**
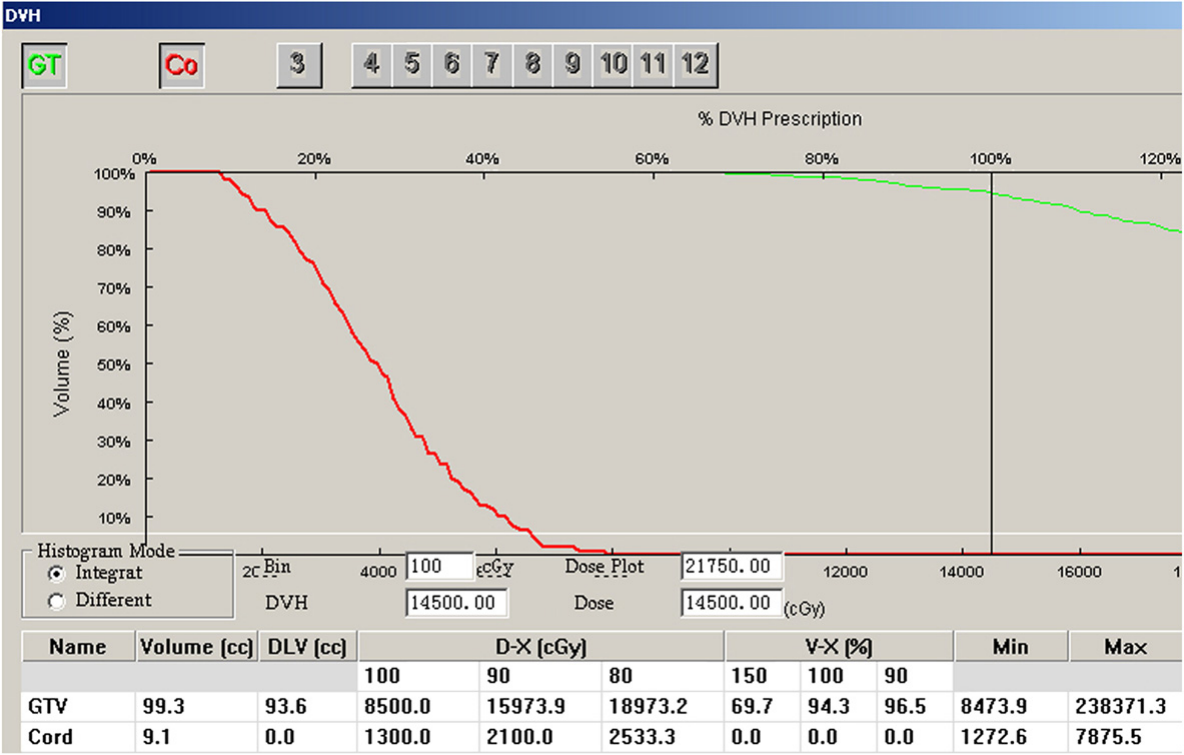
**The Dose volume histograms of GTV and spinal cord after seed implantation.**

From CT images obtained post-seed implant, all ^125^I seed distributions in GTV and 0.5-1.0 cm tumor margin were seen to meet the design requirements. The average number of seeds per patient was 79 (range 7–122), the median specific activity was 0.7 mCi (range 0.5-0.8 mCi) and the D_90_ was 137 Gy (range 90–183 Gy). The exact V_100_ and V_90_ for GTV were showed in Table 3, with prescribed dose 110 Gy. For all the patients, the V_90_ > 90% which was in accordance with our pre-plan. The median maximum dose for spinal cord was 36.3 Gy (range 22–123.2 Gy), for cauda equina, the median maximum dose was 79.5 Gy (range 27–121 Gy). The median maximum dose of esophagus was 28.9 Gy (range 14–42 Gy). The median maximum dose of bowel was 65 Gy (range 62.5-78.2 Gy). Other OARs all meet with the tolerance constraints. At follow-up, all patients were found to have tolerated the procedure, with no need for removal of seeds or cases of pulmonary embolism, and none experienced radiation-induced complications or disease (Table 3, Figure 1, Figure 2).

**Table 3:** **Characteristics of** ^**125**^ **I seed implantation and outcome (n = 17)**

**NO.**	**Lesion size (cm)**	**GTV volume (cc)**	**Seed activity (mCi)/D90 (Gy)**	**GTV:V100/V90**	**Seeds numbers**	**Post-seed implant therapies**	**PS:NRS**	**ASIA**	**Ambulatory function score**	**RR**	**LR (m)**	**Follow-up (m)**	**Cause of death**
1	1.5×1.5×1.0	9.9	0.5/90	92.4/95.2	7	Palliative Surgery	2-3	E		PR	13	69	MM
2	8.5×7.5×7.0	110.2	0.5/100	93.2/96	80	Seed implant	0-1	C		PR	8	11	MM
3	5.5×4.5×11	104.1	0.8/100	90.1/94.2	79	No	5-6	C		SD	39	41	MM
4	2.7×1.5×5.0	102.8	0.8/140	93.2/96.7	92	No	1-2	D		PR	32	32	MM
5	2.5×2.0×1.6	32.6	0.7/121	92.8/94.5	45	Seed implant	0-1	E		CR	34	36	MM
6	5.5×4.1×1.7	103.8	0.72/108	89.2/93.2	109	No	1-2	D		PR	19	19	MM
7	6.2×10.7×8.3	100.3	0.75/148	96.8/99.2	100	CTx	0-1	D		CR	36	36	S
8	8.1×6.3×6.8	99.6	0.73/143	97.5/99	103	No	2	E		PR	35	35	S
9	11.5×3.1×3.5	39.6	0.7/102	87.8/90.7	44	No	0-1	E		PR	21	21	S
10	6.0×3.2×4.1	73.1	0.6/150	99/99.7	122	No	1-2	E		PR	4	4	MM
11	1.4×4.8×3.9	50.5	0.64/164	95.2/95.9	98	No	2	E		PR	17	17	S
12	4.6×3.5×7.7	46.2	0.68/152	98.6/99.3	74	No	0	E		PR	3	3	S
13	4.5×6.4×4.3	27.4	0.7/152	95.1/96.4	47	No	0	E		SD	3	3	S
14	6.0×5.0×4.0	27.8	0.65/133	98.1/99.3	48	No	1	E		PR	12	12	S
15	6.0×3.5×4.5	60.5	0.74/122	93.5/96.2	85	No	3	D		PR	11	11	S
16	2.3×1.8×1.2	22.9	0.75/137	96.3/98.2	31	No	2	D		SD	2	2	S
17	4.8×4.1×5.0	28.2	0.74/183	93.2/96.3	55	Seed implant	0-1	E		SD	14	20	LR

### Local control and survival

3 (17.6%) patients demonstrated tumor recurrence at 8, 14 and 34 months, which received a second round of seed implants. 1 patient died 3 months after the second seed implantation and experienced metastases. The other 2 manifested local survival at the end of follow-up. 6 of the 17 patients (35.3%) died during follow-up and 11 (64.7%) remained alive at the end of follow-up. For 5 chondrosarcomas, the 1-, 2-, 3- year local control rate were 75%, 37.5%, and 37.5%, respectively, with a median of 34 months (range, 4–39 months). For 4 chordomas, the local control rate was 50% with a median follow-up of 13 months (range, 3–17 months). For 3 fibromatosis, all of them were survival without local recurrence at the end of follow-up. And for others with 1 case, the outcomes could be seen in Table 3. All deaths were due to multiple organ failure.

### Pain relief

All patients suffered varying degrees of pain before seed implantation, ranging from 1 to 9 on the NRS. The mean time to pain relief was 2–3 days after the procedure. 1 to 3 months postimplant, all patients experienced easing of pain. Mean NRS scores before and after implantation were 4.48 ± 2.03 and 1.18 ± 1.38, respectively. The efficacy rate was 100% (*P* < 0.05).

### Neurological and ambulatory function

Regarding neurologic function, 11 patients improved according to ASIA grade. 1 grade B became C, 4 grade Cs became Ds, 1 grade C became E and 5 grade Ds became Es. Regarding ambulatory function, 9 patients improved and 2 patients who had been confined to bed before the procedure due to pain recovered sufficiently to undertake daily activities.

### Complications

Only 1 of 17 patients suffered localized bone defects and bone fractures. In this case, before implantation, bone defects can be seen in CT, and after implantation, the lesion size was the same as before, not worsen by radioactive seeds. The mean dose to the involved vertebral body was 121 Gy. 1 developed pitting edema of both lower legs 30 months later. 1 patient complained of pigmentation and desquamation on local skin, corresponding to a grade 1 skin reaction. Such symptoms were relieved by application of trolamine cream (Biafine) for several days.

## Discussion

The gold standard and first line treatment for spinal tumors is surgery, but due to the short expected lifespan of these patients surgical resection has limited clinical benefit. EBRT kills tumor tissue and has advantages of providing pain relief, preventing skeletal related events such as pathologic bone fracture [12], and reducing the size of tumors before surgical resection. Spinal radiosurgery was pioneered by Hamilton et al. in 1995 [13], with the successful treatment of a cohort of five patients. The effects of radiotherapy lag a little behind those of surgery. Generally, it is 10–20 days before pain is reduced or disappears. In addition, EBRT is performed without spinal stabilization. Bone reconstruction should begin 2–4 months after EBRT [14], and this delay of bone remodeling can increase the incidence of vertebral collapse, which in turn leads to pain and symptoms of nerve compression. Furthermore, radiotherapy lacks precision and sometimes less than optimal doses are given to reduce irradiation of the spinal cord [15]. Rogers reported on EBRT combined with brachytherapy that delivered 69.9 Gy to the spinal cord [9], the mean follow-up time was 19.8 months and no patient exhibited radiation myelitis, even with 167.3 Gy. Armstrong treated paraspinal tumors, Pancoast carcinoma and other sarcomas with permanent or temporary brachytherapy, the dose to the spinal cord was 60 Gy, without occurrence of myelitis [16]. Other reported complications include paresthesia, fatigue, dysphagia, nocturia, diarrhea and radiculitis, though these resolve or are rare with modern dose planning and fractionation systems [17]. The dose tolerance for organs at risk could be better contoured.

Vitaz et al. reported that prior adjuvant treatment modalities such as chemotherapy or EBRT typically increase perioperative morbidity and mortality following spinal surgery [18]. The best surgical outcomes are achieved with radical resection of the tumor (removal of the whole spine) [19]. Palliative surgery is less effective, especially in patients with a short survival expectancy according to the Tokuhashi and Tomita spinal tumor grading systems [20-22]. To achieve complete tumor resection, good visualization is of great importance. Open access procedures such as thoracotomy may be used with minimal injury to the surrounding structures and prevention of future spinal instability, but these open approaches can cause additional pain, significant blood loss and require long hospital stays. Kan et al. used video-assisted thoracoscopic surgery to treat primary tumors of the spinal axis, patients with paraspinal neurogenic tumors had an excellent clinical outcome, especially those without intradural extension [23]. Percutaneous vertebroplasty (PVP) is another minimally invasive treatment for spinal malignancies [24,25]. Compared with traditional procedures, PVP is superior for spinal stabilization. Candidates include patients with vertebral hemangioma, vertebral myeloma and vertebral metastases [26,27]. In patients in poor general health, PVP has become an effective option. Pain relief in 24 h has been reported and the result was more than 90% [28-30]. For patients with relatively large scale vertebral destruction or a high incidence of fractures, PVP or PVP with radioactive seed brachytherapy is the preferred treatment [31].

In order to protect normal tissues for repeat irradiation spinal tumors, Memorial Sloan-Kettering Cancer Center was the first in the world who used HDR technique for spinal tumors [32]. Most HDR therapies were operated intraoperatively, for both ^32^P plaques and catheters with ^192^Ir after-loading system. Folkert et al. reported ^32^P for 3 recurrent spinal sarcomas, the local control rate was 62.5% with a mean follow-up of 10.2 months, which is not better than ours (1 case, 35 months after implantation, still survived) [32]. For ^192^Ir HDR in repeat irradiated lesions of spine, another study by Folkert et al. showed 5 patients received single-fraction with a median dose of 14 Gy, 2 patients placed catheters intraoperatively and 3 with image-guided techniques [33]. The median follow-up was 9 months, and at the end of follow-up, no local progression was observed, the pain relief can be gained in 4 patients (80%) 1–4 weeks later. In our study, all patients experienced pain relief at the end of follow-up, the mean time to pain relief was 2–3 days after the procedure. The median D_90_ in our cases was 137 Gy. The ^125^I seeds implantation was minimally invasive, local anesthesia was enough in general, but for HDR general anesthesia was used [33].

^125^I seeds has developed considerably in the past 20 years and plays an important role in the salvage treatment of many malignancies, for both local control and control of metastatic disease [34,35]. With its features of low energy, homogenous dose distribution in the local area, sharp dose gradient between tumor and adjacent normal tissues and protection of organs at risk, interstitial ^125^I seed brachytherapy seems to be an effective salvage approach. CT allows the visualization of small degrees of vertebral destruction, paraspinal soft tissue involvement and the extent of spinal cord invasion [36,37]. The sensitivity and diagnostic accuracy are 66% and 89%, respectively [38]. For spinal tumors, patient selection remains the most important factor in the success of the procedure. CT-guided ^125^I seed brachytherapy is suitable for those who are poor candidates for surgical intervention as assessed by the Enneking or WBB grading system [39], who have recurrent tumor after surgery and EBRT, or who are reluctant to undergo an open surgical procedure or EBRT. Rogers et al. delivered 69.9 Gy to the spinal cord without radiation myelitis, but the recommended clinical dose limit is 45 Gy [9]. Wang et al. reported 19 patients who underwent interstitial ^125^I seed brachytherapy, the median minimal peripheral dose (MPD) was 120 Gy, median follow-up was 22 months. 1-, 2-, 3- and 5-year local control rates were 63%, 47%, 31% and 3%, respectively. 1-, 2-, 3- and 5-year survival rates were 74%, 56%, 43% and 43%, respectively [40]. No myelopathy were encountered. In the present study, neither neurological complications nor radiological abnormalities were recorded at the end of follow-up. The mean KPS was more than 60 before the procedure and all patients had better scores afterwards. Rose et al. found kyphosis to be the most commonly observed clinical deformity secondary to compression fractures and decreased ambulation [41]. In our series, only one patient suffered bone fracture during follow-up. All of them experienced pain relief and 81.8% had an improved ambulation score, which is in accordance with the findings of Rogers, who treated 24 patients with ^125^I seed brachytherapy with a 2 year local control rate and 1 year OS of 87.4% and 40%, respectively, improved ambulation score in 84% of patients and resumed activities in 67% [9]. In our study, 11.8% patients who were confined to bed before the procedure were able to resume activities.

## Conclusion

In the present study, interstitial ^125^I seed brachytherapy appears to achieve a high rate of tumor control and rapid pain relief. This study demonstrated that CT-guided ^125^I seed for recurrent spinal primary tumors is both safe and effective. However, in the case of large volume or irregular shape, the edge of tumors may be overlooked. In addition, the actual seeds number and positions can’t match perfectly with preoperative plan due to personal equation, which leads to great importance of dose verification [42]. Therefore, strict adherence to the scientific method remains crucial. Our study appears lack of controlled group and long term follow-up results, in order to determine the indications, risks and benefits of ^125^I seed brachytherapy compared with more conventional approaches, large numbers of patients and prospective studies are needed.

## Abbreviations

CT: Computed tomography
EBRT: External beam radiotherapy
DVH: Dose Volume Histogram
D_90_: Prescribed Dose Delivered to 90% of the Gross Tumor Volume
KPS: Karnofsky performance status
GTV: Gross tumor volume
PTV: Planning target volume
3D: Three-dimensional
OS: Overall survival
NRS: Numeric rating scale
ASIA: American Spinal Injury Association
PVP: Percutaneous vertebroplasty
TPS: Treatment Planning System
WBB: Weinstein Boriani Biagini
MPD: Median minimal peripheral dose
PS: Pain Score
S: Surgery
RT: Radiotherapy
CTx: Chemotherapy
RR: Response rate
PR: Partial Response
CR: Complete Response
SD: Stable Disease
MM: Multiple metastasis
m: Months
LR: Local recurrence
S: Survival
